# (*Z*)-2-(4-Chloro­benzyl­idene)benzo[*d*]thia­zolo[3,2-*a*]imidazol-3(2*H*)-one

**DOI:** 10.1107/S1600536812015516

**Published:** 2012-04-18

**Authors:** Hoong-Kun Fun, Suchada Chantrapromma, Hatem A. Abdel-Aziz

**Affiliations:** a X-ray Crystallography Unit, School of Physics, Universiti Sains Malaysia, 11800 USM, Penang, Malaysia; b Crystal Materials Research Unit, Department of Chemistry, Faculty of Science, Prince of Songkla University, Hat-Yai, Songkhla 90112, Thailand; c Department of Pharmaceutical Chemistry, College of Pharmacy, King Saud University, PO Box 2457, Riyadh 11451, Saudi Arabia

## Abstract

The mol­ecule of the title compound, C_16_H_9_ClN_2_OS, is approximately planar, the dihedral angle between the thia­zolo[3,2-*a*]benzimidazole ring system and the 4-chloro­phenyl ring being 2.10 (5)°. An intra­molecular C—H⋯S inter­action generates an *S*(6) ring motif. In the crystal, mol­ecules are stacked into columns along the *b* axis by π–π inter­actions with centroid–centroid distances of 3.6495 (7)–3.9546 (8) Å.

## Related literature

For bond-length data, see: Allen *et al.* (1987 ▶). For hydrogen-bond motifs, see: Bernstein *et al.* (1995 ▶). For background to and the biological activity of thia­zolo[3,2-*a*]benzimidazoles, see: Abdel-Aziz, El-Zahabi & Dawood (2010 ▶); Abdel-Aziz, Hamdy *et al.* (2007 ▶, 2008 ▶); Abdel-Aziz, Saleh & El-Zahabi (2010 ▶); Al-Rashood & Abdel-Aziz (2010 ▶); Chimirri *et al.* (1988 ▶); Farag *et al.* (2011 ▶); Hamdy *et al.* (2007 ▶); Mavrova *et al.* (2005 ▶). For the stability of the temperature controller, see: Cosier & Glazer (1986 ▶).
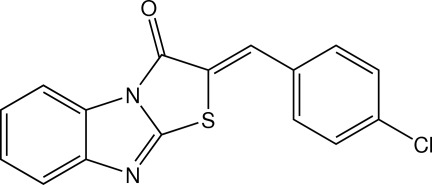


## Experimental

### 

#### Crystal data

C_16_H_9_ClN_2_OS
*M* *_r_* = 312.77
Triclinic, 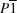

*a* = 7.0182 (4) Å
*b* = 7.3443 (4) Å
*c* = 13.7142 (8) Å
α = 91.742 (1)°
β = 100.836 (1)°
γ = 112.878 (1)°
*V* = 635.47 (6) Å^3^
*Z* = 2
Mo *K*α radiation
μ = 0.46 mm^−1^
*T* = 100 K
0.37 × 0.18 × 0.06 mm

#### Data collection

Bruker APEX DUO CCD area-detector diffractometer
Absorption correction: multi-scan (*SADABS*; Bruker, 2009 ▶) *T*_min_ = 0.848, *T*_max_ = 0.973
14145 measured reflections
3660 independent reflections
3233 reflections with *I* > 2σ(*I*)
*R*_int_ = 0.026

#### Refinement

*R*[*F* ^2^ > 2σ(*F* ^2^)] = 0.030
*wR*(*F* ^2^) = 0.082
*S* = 1.05
3660 reflections
190 parameters
H-atom parameters constrained
Δρ_max_ = 0.56 e Å^−3^
Δρ_min_ = −0.27 e Å^−3^

### 

Data collection: *APEX2* (Bruker, 2009 ▶); cell refinement: *SAINT* (Bruker, 2009 ▶); data reduction: *SAINT*; program(s) used to solve structure: *SHELXTL* (Sheldrick, 2008 ▶); program(s) used to refine structure: *SHELXTL*; molecular graphics: *SHELXTL*; software used to prepare material for publication: *SHELXTL* and *PLATON* (Spek, 2009 ▶).

## Supplementary Material

Crystal structure: contains datablock(s) global, I. DOI: 10.1107/S1600536812015516/is5117sup1.cif

Structure factors: contains datablock(s) I. DOI: 10.1107/S1600536812015516/is5117Isup2.hkl

Supplementary material file. DOI: 10.1107/S1600536812015516/is5117Isup3.cml

Additional supplementary materials:  crystallographic information; 3D view; checkCIF report

## Figures and Tables

**Table 1:** Hydrogen-bond geometry (Å, °)

*D*—H⋯*A*	*D*—H	H⋯*A*	*D*⋯*A*	*D*—H⋯*A*
C16—H16*A*⋯S1	0.93	2.50	3.2161 (13)	133

**Fig. 1.**
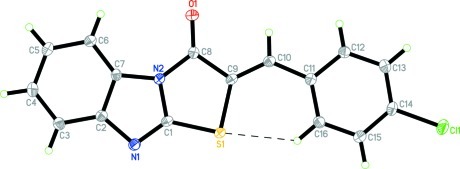
The molecular structure of the title compound with 50% probability displacement ellipsoids and the atom-numbering scheme. Intramolecular C—H···S weak interaction was shown as dashed line.

**Fig. 2.**
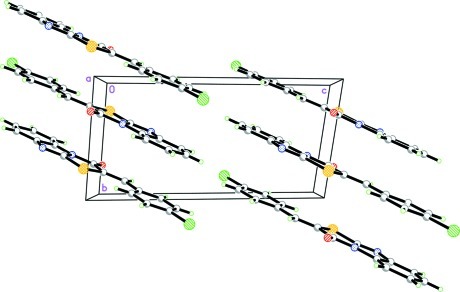
The crystal packing of the title compound viewed along the a axis.

## supplementary crystallographic information

### Comment

There are considerable interest in the chemistry of thiazolo[3,2-*a*]benzimidazoles and their unique pharmaceutical and medicinal applications have been reported. These activities including antibacterial, antifungal, anti-inflammatory, antiulcer, antiviral, anthelmintic and anticancer properties (Al-Rashood *et al.*, 2010; Chimirri *et al.*, 1988). The parasitological study in vitro has also shown that the analogs of the title compound exhibited higher activity than albendazole against *T. spiralis* (Mavrova *et al.*, 2005). These considerable biological activities as well as in continuation of our interests in the chemistry and biological activities of these compounds (Abdel-Aziz, Hamdy *et al.*, 2007, 2008; Abdel-Aziz, Saleh & El-Zahabi, 2010; Farag *et al.*, 2011; Hamdy *et al.*, 2007) have lead us to synthesize and present the X-ray structural analysis of the title compound (I).

In the molecular structure of (I), C_14_H_11_ClN_4_O_4_, the thiazolo[3,2-*a*]benzimidazole ring system is planar with an *r.m.s*. deviation 0.019 (12) Å for the thirteen non H-atoms (C1–C9/N1/N2/O1/S1) and the 4-chlorobenzilidene unit is also planar with an *r.m.s* deviation 0.002 (12) Å for the eight non H-atoms (C10–C16/Cl1). The dihedral between the mean plane through the thiazolo[3,2-*a*]benzimidazole ring system and 4-chlorophenyl ring is 2.10 (5)°. An intramolecular C—H···S weak interaction (Fig. 1 and Table 1) generates an S(6) ring motif (Bernstein *et al.*, 1995) which help to stabilize the planarity of the molecule. The bond distances agree with the literature values (Allen *et al.*, 1987).

In the crystal packing (Fig. 2), the molecules are stacked into column along the *b* axis by π–π interactions with the distances of *Cg*1···*Cg*1^i^ = 3.8297 (7) Å, *Cg*2···*Cg*4^ii^ = 3.9545 (8) Å, *Cg*3···*Cg*4^i^ = 3.7691 (8) Å and *Cg*3···*Cg*4^ii^ = 3.6495 (7) Å [symmetry codes: (i) 1-x, 1-y, -z; (ii) 1-x, -y, -z]. *Cg*1, *Cg*2, *Cg*3 and *Cg*4 are the centroids of S1/C1/N2/C8/C9, C1/C2/C7/N1/N2, C2–C7 and C11–C16 rings, respectively.

### Experimental

The one-pot synthesis of the title compound was carried out by a cyclocondensation of 2-mercaptobenzimidazole, chloroacetic acid, 4-chloro benzaldehyde, acetic anhydride and glacial acetic acid in the presence of sodium acetate to afford the title compound (Mavrova *et al.*, 2005; Abdel-Aziz, El-Zahabi & Dawood, 2010). Yellow needle-shaped single crystals of the title compound suitable for *x*-ray structure determination were recrystalized from ethanol by slow evaporation of the solvent at room temperature over several days.

### Refinement

All H atoms were positioned geometrically and allowed to ride on their parent atoms, with d(C—H) = 0.93 Å for aromatic and CH atoms, and the *U*_iso_(H) values were constrained to be 1.2*U*_eq_ of the carrier atoms

### Crystal data

**Table:**

C_16_H_9_ClN_2_OS	*Z* = 2
*M**_r_* = 312.77	*F*(000) = 320
Triclinic, *P*1	*D*_x_ = 1.635 Mg m^−^^3^
Hall symbol: -P 1	Mo *K*α radiation, λ = 0.71073 Å
*a* = 7.0182 (4) Å	Cell parameters from 3660 reflections
*b* = 7.3443 (4) Å	θ = 1.5–30.0°
*c* = 13.7142 (8) Å	µ = 0.46 mm^−^^1^
α = 91.742 (1)°	*T* = 100 K
β = 100.836 (1)°	Needle, yellow
γ = 112.878 (1)°	0.37 × 0.18 × 0.06 mm
*V* = 635.47 (6) Å^3^	

### Data collection

**Table:**

Bruker APEX DUO CCD area-detector diffractometer	3660 independent reflections
Radiation source: sealed tube	3233 reflections with *I* > 2σ(*I*)
Graphite monochromator	*R*_int_ = 0.026
φ and ω scans	θ_max_ = 30.0°, θ_min_ = 1.5°
Absorption correction: multi-scan (*SADABS*; Bruker, 2009)	*h* = −9→9
*T*_min_ = 0.848, *T*_max_ = 0.973	*k* = −10→10
14145 measured reflections	*l* = −19→19

### Refinement

**Table:**

Refinement on *F*^2^	Primary atom site location: structure-invariant direct methods
Least-squares matrix: full	Secondary atom site location: difference Fourier map
*R*[*F*^2^ > 2σ(*F*^2^)] = 0.030	Hydrogen site location: inferred from neighbouring sites
*wR*(*F*^2^) = 0.082	H-atom parameters constrained
*S* = 1.05	*w* = 1/[σ^2^(*F*_o_^2^) + (0.0366*P*)^2^ + 0.3855*P*] where *P* = (*F*_o_^2^ + 2*F*_c_^2^)/3
3660 reflections	(Δ/σ)_max_ = 0.001
190 parameters	Δρ_max_ = 0.56 e Å^−^^3^
0 restraints	Δρ_min_ = −0.27 e Å^−^^3^

### Special details

**Table:**

Experimental. The crystal was placed in the cold stream of an Oxford Cryosystems Cobra open-flow nitrogen cryostat (Cosier & Glazer, 1986) operating at 100.0 (1) K.
Geometry. All esds (except the esd in the dihedral angle between two l.s. planes) are estimated using the full covariance matrix. The cell esds are taken into account individually in the estimation of esds in distances, angles and torsion angles; correlations between esds in cell parameters are only used when they are defined by crystal symmetry. An approximate (isotropic) treatment of cell esds is used for estimating esds involving l.s. planes.
Refinement. Refinement of F^2^ against ALL reflections. The weighted R-factor wR and goodness of fit S are based on F^2^, conventional R-factors R are based on F, with F set to zero for negative F^2^. The threshold expression of F^2^ > 2sigma(F^2^) is used only for calculating R-factors(gt) etc. and is not relevant to the choice of reflections for refinement. R-factors based on F^2^ are statistically about twice as large as those based on F, and R- factors based on ALL data will be even larger.

### Fractional atomic coordinates and isotropic or equivalent isotropic displacement parameters (Å^2^)

**Table:**

	*x*	*y*	*z*	*U*_iso_*/*U*_eq_	
S1	0.34693 (4)	0.25459 (4)	0.03735 (2)	0.01299 (8)	
Cl1	−0.35675 (5)	−0.18707 (5)	−0.43869 (2)	0.02057 (9)	
O1	0.88271 (14)	0.27130 (14)	−0.01062 (7)	0.01625 (18)	
N1	0.58054 (16)	0.43157 (15)	0.22785 (8)	0.0138 (2)	
N2	0.75134 (15)	0.36857 (15)	0.11464 (7)	0.01179 (19)	
C1	0.56374 (18)	0.36129 (17)	0.13730 (9)	0.0123 (2)	
C2	0.79803 (18)	0.49233 (17)	0.27094 (9)	0.0125 (2)	
C3	0.90598 (19)	0.57701 (18)	0.36781 (9)	0.0147 (2)	
H3A	0.8365	0.6030	0.4144	0.018*	
C4	1.1227 (2)	0.62158 (18)	0.39238 (9)	0.0156 (2)	
H4A	1.1988	0.6788	0.4566	0.019*	
C5	1.22857 (19)	0.58244 (18)	0.32293 (9)	0.0149 (2)	
H5A	1.3731	0.6138	0.3422	0.018*	
C6	1.12230 (18)	0.49741 (18)	0.22550 (9)	0.0135 (2)	
H6A	1.1918	0.4713	0.1789	0.016*	
C7	0.90738 (18)	0.45404 (17)	0.20197 (9)	0.0117 (2)	
C8	0.73740 (18)	0.28632 (17)	0.01951 (9)	0.0121 (2)	
C9	0.51258 (18)	0.21858 (17)	−0.03702 (9)	0.0120 (2)	
C10	0.45896 (18)	0.14096 (17)	−0.13349 (9)	0.0129 (2)	
H10A	0.5708	0.1362	−0.1591	0.015*	
C11	0.25559 (18)	0.06330 (17)	−0.20435 (9)	0.0125 (2)	
C12	0.25076 (19)	−0.00568 (18)	−0.30184 (9)	0.0140 (2)	
H12A	0.3757	0.0004	−0.3181	0.017*	
C13	0.0643 (2)	−0.08267 (18)	−0.37443 (9)	0.0154 (2)	
H13A	0.0636	−0.1272	−0.4388	0.018*	
C14	−0.12171 (19)	−0.09177 (18)	−0.34886 (9)	0.0144 (2)	
C15	−0.12333 (19)	−0.02595 (18)	−0.25342 (9)	0.0149 (2)	
H15A	−0.2493	−0.0337	−0.2377	0.018*	
C16	0.06430 (19)	0.05172 (18)	−0.18134 (9)	0.0141 (2)	
H16A	0.0635	0.0965	−0.1173	0.017*	

### Atomic displacement parameters (Å^2^)

**Table:**

	*U*^11^	*U*^22^	*U*^33^	*U*^12^	*U*^13^	*U*^23^
S1	0.01026 (13)	0.01520 (14)	0.01318 (14)	0.00536 (10)	0.00154 (10)	−0.00024 (10)
Cl1	0.01474 (14)	0.02600 (17)	0.01591 (15)	0.00557 (12)	−0.00226 (11)	−0.00026 (11)
O1	0.0137 (4)	0.0197 (4)	0.0166 (4)	0.0080 (3)	0.0038 (3)	−0.0002 (3)
N1	0.0123 (4)	0.0143 (5)	0.0146 (5)	0.0056 (4)	0.0021 (4)	0.0006 (4)
N2	0.0103 (4)	0.0130 (4)	0.0123 (4)	0.0052 (3)	0.0018 (3)	0.0012 (4)
C1	0.0108 (5)	0.0127 (5)	0.0145 (5)	0.0056 (4)	0.0029 (4)	0.0021 (4)
C2	0.0126 (5)	0.0113 (5)	0.0140 (5)	0.0056 (4)	0.0024 (4)	0.0015 (4)
C3	0.0163 (5)	0.0142 (5)	0.0136 (5)	0.0065 (4)	0.0029 (4)	0.0007 (4)
C4	0.0176 (5)	0.0135 (5)	0.0134 (5)	0.0057 (4)	−0.0007 (4)	0.0004 (4)
C5	0.0129 (5)	0.0139 (5)	0.0163 (6)	0.0050 (4)	0.0001 (4)	0.0021 (4)
C6	0.0124 (5)	0.0131 (5)	0.0153 (5)	0.0056 (4)	0.0028 (4)	0.0021 (4)
C7	0.0131 (5)	0.0108 (5)	0.0108 (5)	0.0050 (4)	0.0013 (4)	0.0013 (4)
C8	0.0125 (5)	0.0104 (5)	0.0126 (5)	0.0044 (4)	0.0016 (4)	0.0013 (4)
C9	0.0096 (5)	0.0112 (5)	0.0155 (5)	0.0047 (4)	0.0023 (4)	0.0023 (4)
C10	0.0125 (5)	0.0121 (5)	0.0149 (5)	0.0059 (4)	0.0029 (4)	0.0024 (4)
C11	0.0130 (5)	0.0106 (5)	0.0135 (5)	0.0047 (4)	0.0017 (4)	0.0017 (4)
C12	0.0141 (5)	0.0141 (5)	0.0143 (5)	0.0062 (4)	0.0030 (4)	0.0013 (4)
C13	0.0176 (5)	0.0152 (5)	0.0125 (5)	0.0064 (4)	0.0020 (4)	0.0008 (4)
C14	0.0136 (5)	0.0125 (5)	0.0142 (5)	0.0042 (4)	−0.0009 (4)	0.0016 (4)
C15	0.0129 (5)	0.0158 (5)	0.0161 (6)	0.0059 (4)	0.0033 (4)	0.0027 (4)
C16	0.0148 (5)	0.0144 (5)	0.0131 (5)	0.0061 (4)	0.0028 (4)	0.0010 (4)

### Geometric parameters (Å, º)

**Table:**

S1—C1	1.7411 (12)	C6—C7	1.3849 (16)
S1—C9	1.7692 (12)	C6—H6A	0.9300
Cl1—C14	1.7363 (12)	C8—C9	1.4981 (16)
O1—C8	1.2108 (14)	C9—C10	1.3480 (16)
N1—C1	1.2967 (15)	C10—C11	1.4556 (16)
N1—C2	1.4119 (15)	C10—H10A	0.9300
N2—C1	1.3903 (14)	C11—C12	1.4049 (16)
N2—C8	1.3909 (15)	C11—C16	1.4076 (16)
N2—C7	1.3980 (15)	C12—C13	1.3871 (17)
C2—C3	1.3895 (16)	C12—H12A	0.9300
C2—C7	1.4097 (16)	C13—C14	1.3917 (17)
C3—C4	1.3955 (17)	C13—H13A	0.9300
C3—H3A	0.9300	C14—C15	1.3850 (17)
C4—C5	1.3990 (17)	C15—C16	1.3888 (17)
C4—H4A	0.9300	C15—H15A	0.9300
C5—C6	1.3942 (17)	C16—H16A	0.9300
C5—H5A	0.9300		
			
C1—S1—C9	90.12 (5)	O1—C8—C9	126.82 (11)
C1—N1—C2	103.32 (10)	N2—C8—C9	107.93 (10)
C1—N2—C8	116.71 (10)	C10—C9—C8	119.68 (10)
C1—N2—C7	105.85 (9)	C10—C9—S1	128.06 (9)
C8—N2—C7	137.35 (10)	C8—C9—S1	112.26 (8)
N1—C1—N2	115.19 (10)	C9—C10—C11	130.90 (11)
N1—C1—S1	131.92 (9)	C9—C10—H10A	114.5
N2—C1—S1	112.88 (9)	C11—C10—H10A	114.5
C3—C2—C7	120.04 (11)	C12—C11—C16	118.15 (11)
C3—C2—N1	128.60 (11)	C12—C11—C10	117.52 (10)
C7—C2—N1	111.36 (10)	C16—C11—C10	124.33 (11)
C2—C3—C4	117.38 (11)	C13—C12—C11	121.66 (11)
C2—C3—H3A	121.3	C13—C12—H12A	119.2
C4—C3—H3A	121.3	C11—C12—H12A	119.2
C3—C4—C5	121.78 (11)	C12—C13—C14	118.53 (11)
C3—C4—H4A	119.1	C12—C13—H13A	120.7
C5—C4—H4A	119.1	C14—C13—H13A	120.7
C6—C5—C4	121.47 (11)	C15—C14—C13	121.46 (11)
C6—C5—H5A	119.3	C15—C14—Cl1	119.27 (9)
C4—C5—H5A	119.3	C13—C14—Cl1	119.26 (9)
C7—C6—C5	116.23 (11)	C14—C15—C16	119.61 (11)
C7—C6—H6A	121.9	C14—C15—H15A	120.2
C5—C6—H6A	121.9	C16—C15—H15A	120.2
C6—C7—N2	132.61 (11)	C15—C16—C11	120.58 (11)
C6—C7—C2	123.10 (11)	C15—C16—H16A	119.7
N2—C7—C2	104.28 (10)	C11—C16—H16A	119.7
O1—C8—N2	125.24 (11)		
			
C2—N1—C1—N2	0.05 (14)	C1—N2—C8—O1	−176.11 (12)
C2—N1—C1—S1	179.02 (10)	C7—N2—C8—O1	−0.2 (2)
C8—N2—C1—N1	177.22 (10)	C1—N2—C8—C9	3.12 (14)
C7—N2—C1—N1	0.13 (14)	C7—N2—C8—C9	178.99 (13)
C8—N2—C1—S1	−1.95 (13)	O1—C8—C9—C10	−3.77 (19)
C7—N2—C1—S1	−179.04 (8)	N2—C8—C9—C10	177.02 (11)
C9—S1—C1—N1	−178.97 (13)	O1—C8—C9—S1	176.23 (11)
C9—S1—C1—N2	0.01 (9)	N2—C8—C9—S1	−2.99 (12)
C1—N1—C2—C3	−179.20 (12)	C1—S1—C9—C10	−178.28 (12)
C1—N1—C2—C7	−0.20 (13)	C1—S1—C9—C8	1.72 (9)
C7—C2—C3—C4	0.23 (18)	C8—C9—C10—C11	179.37 (11)
N1—C2—C3—C4	179.15 (12)	S1—C9—C10—C11	−0.6 (2)
C2—C3—C4—C5	−0.26 (18)	C9—C10—C11—C12	178.99 (12)
C3—C4—C5—C6	0.22 (19)	C9—C10—C11—C16	−1.5 (2)
C4—C5—C6—C7	−0.14 (18)	C16—C11—C12—C13	0.28 (18)
C5—C6—C7—N2	−179.28 (12)	C10—C11—C12—C13	179.85 (11)
C5—C6—C7—C2	0.12 (18)	C11—C12—C13—C14	−0.30 (19)
C1—N2—C7—C6	179.25 (13)	C12—C13—C14—C15	0.04 (19)
C8—N2—C7—C6	3.1 (2)	C12—C13—C14—Cl1	179.84 (9)
C1—N2—C7—C2	−0.24 (12)	C13—C14—C15—C16	0.24 (19)
C8—N2—C7—C2	−176.40 (13)	Cl1—C14—C15—C16	−179.57 (9)
C3—C2—C7—C6	−0.17 (18)	C14—C15—C16—C11	−0.26 (19)
N1—C2—C7—C6	−179.26 (11)	C12—C11—C16—C15	0.01 (18)
C3—C2—C7—N2	179.38 (11)	C10—C11—C16—C15	−179.53 (11)
N1—C2—C7—N2	0.28 (13)		

### Hydrogen-bond geometry (Å, º)

**Table:**

*D*—H···*A*	*D*—H	H···*A*	*D*···*A*	*D*—H···*A*
C16—H16*A*···S1	0.93	2.50	3.2161 (13)	133
